# Identification of cis-regulatory motifs in first introns and the prediction of intron-mediated enhancement of gene expression in *Arabidopsis thaliana*

**DOI:** 10.1186/s12864-021-07711-1

**Published:** 2021-05-26

**Authors:** Georg Back, Dirk Walther

**Affiliations:** Max Planck Institute of Molecular Plant Physiology, 14476 Potsdam, Germany

**Keywords:** Gene expression, Introns, Intron-mediated enhancement, Sequence motifs, Random forests, *Arabidopsis thaliana*

## Abstract

**Background:**

Intron mediated enhancement (IME) is the potential of introns to enhance the expression of its respective gene. This essential function of introns has been observed in a wide range of species, including fungi, plants, and animals. However, the mechanisms underlying the enhancement are as of yet poorly understood. The goal of this study was to identify potential IME-related sequence motifs and genomic features in first introns of genes in *Arabidopsis thaliana*.

**Results:**

Based on the rationale that functional sequence motifs are evolutionarily conserved, we exploited the deep sequencing information available for *Arabidopsis thaliana*, covering more than one thousand Arabidopsis accessions, and identified 81 candidate hexamer motifs with increased conservation across all accessions that also exhibit positional occurrence preferences. Of those, 71 were found associated with increased correlation of gene expression of genes harboring them, suggesting a cis-regulatory role. Filtering further for effect on gene expression correlation yielded a set of 16 hexamer motifs, corresponding to five consensus motifs. While all five motifs represent new motif definitions, two are similar to the two previously reported IME-motifs, whereas three are altogether novel. Both consensus and hexamer motifs were found associated with higher expression of alleles harboring them as compared to alleles containing mutated motif variants as found in naturally occurring Arabidopsis accessions. To identify additional IME-related genomic features, Random Forest models were trained for the classification of gene expression level based on an array of sequence-related features. The results indicate that introns contain information with regard to gene expression level and suggest sequence-compositional features as most informative, while position-related features, thought to be of central importance before, were found with lower than expected relevance.

**Conclusions:**

Exploiting deep sequencing and broad gene expression information and on a genome-wide scale, this study confirmed the regulatory role on first-introns, characterized their intra-species conservation, and identified a set of novel sequence motifs located in first introns of genes in the genome of the plant *Arabidopsis thaliana* that may play a role in inducing high and correlated gene expression of the genes harboring them.

**Supplementary Information:**

The online version contains supplementary material available at 10.1186/s12864-021-07711-1.

## Introduction

Introns, seemingly superfluous intragenic regions, are found across almost all species, in particular in eukaryotes [1]. The question as to which functions introns and intron splicing have has been discussed since their discovery. Their almost universal occurrence seems to suggest that introns play an essential role. Allowing alternative splicing that leads to an expansion of the protein repertoire of organisms and thus increased complexity and phenotypic diversity [2] is one of the leading explanations for the prevalence of introns. Besides alternative splicing, mRNA-stability has been linked to introns as splicing was found associated with increased mRNA half-life [3]. Specifically, splicing can assist in the 3′-end formation of mRNAs by recruiting capping factors [4]. Furthermore, introns can contain RNA genes, such as snoRNAs, long non-coding RNAs (lncRNAs), miRNAs, and small-interfering RNAs (siRNAs) [1]. Those intron-located RNAs can exert regulatory roles on their host genes [5].

As perhaps one of the most essential functions of introns, the enhancement of gene expression has been reported. Studies have shown that certain introns are able to enhance the expression of their respective genes by a significant amount [6, 7]. Interestingly, and in contrast to regular enhancer elements, these introns have to be transcribed to trigger this effect [8]. This enhancement, known as Intron Mediated Enhancement (IME), is even strong enough to be used as a tool in the repertoire of molecular biology techniques to boost the expression of specific target genes, and has been suggested to contribute to the high expression levels of housekeeping genes [9]. IME was one of the earliest surmised functions of introns, when it was discovered in 1987 in maize [10]. Since then, IME has been found in a variety of species, from plants to vertebrates and nematodes [11, 12]. It has been reported that IME can act via increased transcription rate, increased nuclear export of the transcript, increased transcript stability, and even enhanced translation efficiency [13, 14]. The mechanisms responsible for these diverse modes of action of introns on the gene expression are not yet understood. However, a strong correlation between the proximity of an intron to the transcription start site (TSS) and its potential to enhance expression has been observed, with the vast majority of reported IME found associated with the first (5′-most) intron of a gene [15]. Furthermore, both splicing-dependent and splicing-independent effects have been reported [6, 9, 16].

Primarily, IME-introns have been identified by experimental evidence [10, 17, 18]. While this is essential for gaining further insight into IME, the currently known set may cover only a small portion of all IME introns. To identify IME introns on a larger scale, bioinformatic methods are required. Currently, IMEter is the only available computational method for IME-intron detection, which works under the assumption that TSS-proximal introns are enriched in IME sequence motifs assumed as words (k-mers of length 5) [19, 20]. IMEter computes a log-odds score for an intron sequence to correspond to TSS-proximal and, hence, IME-signal-bearing introns by scoring the present pentamers relative to observed average relative frequencies of pentamers in TSS-proximal vs. TSS-distal introns. This straightforward approach has yielded promising results. Many of the previously established IME-introns were assigned high scores by IMEter [21]. Furthermore, in top scoring introns, two sequence motifs were detected, which, when present at high densities, are able to induce IME [17, 21]. These motifs even led to an increase of mRNA levels when located within exons [9]. However, not all introns, reported to induce IME, score accordingly with IMEter or are enriched for the two reported motifs [9, 21]. Therefore, alternative computational approaches may identify additional regulatory motifs in introns.

Phylogenetic footprinting, a commonly used strategy to bioinformatically identify functional genome sequence motifs, assumes that functional motifs are conserved across different species. With available sequence and associated single nucleotide polymorphism (SNP) information, this approach can also be applied to intra-species evolution, as applied, for example, in *Arabidopsis thaliana* [22]. Here, a large set of genome sequences is essential to include sufficient sequence divergence in order to achieve a high motif resolution. The 1001-Arabidopsis-genome-project provides such data that includes a Single Nucleotide Polymorphism (SNP) set for 1135 fully sequenced *Arabidopsis thaliana* accessions [23]. Moreover, a large compendium of gene expression data (microarray- and RNA-seq-based) is available, allowing to test whether introns sharing a particular motif also share a similar expression pattern as well as available methylome data, permitting to include epigenetic information in the analysis [24]. A previous study succeeded in identifying novel motifs in promoter regions using the 1001-genome project SNP set and available expression information [25]. The authors compared sequence conservation not only across single motif mapping locations, but compared all mapping locations of a given motif. This approach circumvents the problem of the relatively low SNP density across the Arabidopsis accessions by determining the degree of conservation of a motif over all its occurrences in the genome.

The present study builds on the rationale that IME-motifs are conserved more than expected by chance and uses a SNP-based approach to identify cis-regulatory intron-located elements, initially defined as sequence hexamers. By adding conservation and location distribution as characteristic features associated with IME candidate motifs, our approach attempts to extend the concepts established by IMEter, which relies on candidate motif occurrence differences in the first vs. other introns alone. Differential methylation as a potential regulator of IME was also investigated here. For validation of functional relevance, correlation of gene expression of all genes containing candidate IME motifs in their first intron was used. In addition, we tested the effect of mutations on the activity of candidate IME-motifs by exploiting the naturally occurring variation in the different Arabidopsis accessions along with associated RNAseq-based expression information.

To assess the information contents of intronic sequences on gene expression and to extract associated informative features, this study also includes a Random Forest (RF) classification model for the prediction of mRNA expression levels based on intron sequence information. A number of sequence characteristics of the respective first intron, such as intron length, nucleotide composition, distance to TSS, distance to the translation start codon, and the IMEter score served as features for the Random Forest classifier. In addition, folding energetics of intronic RNA, cross-species conservation, and presence of transposons was considered as well. The goal was not only to create an accurate model, but also to extract features that contribute to the prediction accuracy in addition to the more targeted k-mer motif approach.

We report the identification of 16 candidate IME motifs, collapsing to five consensus motifs. While all five motifs constitute new motif definitions, two resemble previously reported IMEter motifs, and three appear altogether novel. The RF-models confirm the predictive potential of introns with regard to the expression level of their host genes and suggest features associated with base composition as particularly informative. In sum, our results shed new light on the possible mode of action responsible for IME and may serve as a starting point for further approaches examining IME in the future.

## Materials and methods

### Extraction of intron positions and sequences

Version 10 of the Arabidopsis Information Resource (TAIR) [26] General Feature Format version 3 (GFF3) file was used to extract the sequence coordinates of all mRNA introns within the *Arabidopsis thaliana* genome sequence via exon positions to infer intron positions. All introns shorter than ten base pairs (bp) were excluded. A FASTA file containing all introns was created by using bedtools [27] and the complete TAIR10 genome sequence as a reference. The intron set was then split into first, i.e. the promoter-proximal intron set, and the set of other introns. Introns located in the 5’UTR of a gene were detected by an overlap between an artificially length-extended (5 bp at either end) intron and 5’UTR coordinates.

### Extraction of relevant single nucleotide polymorphisms (SNPs)

SNPs were extracted from the 1001 Arabidopsis genome project variance calling file (VCF) [23]. All variants that were positioned in one of the introns were extracted. A threshold of 50 was set as the minor allele frequency for SNP positions to be considered and 500 valid (i.e. non-“N”) alleles called, with alleles counted as haploid counts (i.e. counts per chromosome). With VCFtools [28], the resulting VCF file was used to extract all SNP positions. In total, 2,426,458 SNPs were used, of which 382,016 were located in introns.

### Selection of candidate hexamers

#### Selection of k-mer size

As a compromise between specificity of motifs (favoring longer motifs) and the combinatorial increase associated with increasing motif-length, a k-mer size of k = 6 was chosen, from here on termed hexamers. For each hexamer, their respective positions in each intron were determined using the extracted intron sequences. To avoid a bias towards hexamers containing part of the highly conserved splice sites, the first and last three sequence positions of each intron were excluded from the analysis. From the obtained hexamer positions, the frequency and distribution of hexamers within the introns were determined. For analyzing conservation, frequency, and location distribution, results for reverse-complementary hexamers were combined with their forward definitions and treated as one hexamer.

#### Relative frequency of hexamers

Similar to IMEter [21], the frequency of hexamers in first introns compared to other introns was taken as the initial criterion for the identification of potential regulatory hexamers. For both intron sets, first and other introns, the total occurrence of each hexamer, H_i_, over all introns in the Col-0 reference genome sequence was determined, and then normalized by the total occurrence of all hexamers for each intron group, respectively. Afterwards, the relative frequency, F, was calculated by dividing the normalized frequency of hexamers in the first by the normalized frequency of hexamers in the other introns, with

1 $$ {F}_{{\mathrm{H}}_i}=\frac{C_{f,{H}_i}/{\sum}_{j=1}^N{C}_{f,{H}_j}}{C_{o,{H}_i}/{\sum}_{j=1}^N{C}_{o,{H}_j}}, $$

where C stands for counts, H for hexamer, f and o for first and others, respectively. N is the total number of observed hexamers (*N* = 2080).

#### Degree of conservation of hexamers, conservation rate

To assess the degree of conservation of each hexamer, the total number of occurrences of each hexamer introns was compared to the occurrence of the same hexamer with SNP positions masked, performed separately for first and other introns. The masking was done by replacing each position containing a SNP with a symbol not used in the nucleic acid notation, here “*”. The degree of conservation was calculated as the ratio of hexamer counts, C_H_, with SNPs masked and the counts without masking. This provides a position and alignment independent measure of conservation with ratio-values near one suggesting high conservation and smaller ratios suggesting increasing variability. For comparison, the randomly expected conservation was computed as

2 $$ {C}_r={\left(1-\frac{N_{SNP}}{N_{bp}}\right)}^k, $$

where N_SNP_ is the number of SNP-positions found in introns and N_bp_ is the total number of positions in respective introns, computed separately for first and other introns. C_r_ corresponds to the probability of a k-mer not containing any SNP position given the background SNP-density.

#### Positional distribution of hexamers in introns

Two factors were considered for the location distribution of hexamers within introns. First, since many cis-regulatory elements show preferences for specific localization, we hypothesized that relevant hexamers should show a characteristic distribution, which significantly differs from a uniform distribution. To examine this, the relative positioning of each occurrence of a hexamer in an intron was determined by dividing the first position of each hexamer occurrence by the length of the respective intron. These relative start positions were then binned into ten bins covering an interval of (0, 1). Based on the binned occurrence counts, positional preferences were expressed as position entropies, S_H_, with

3 $$ {S}_H=-{\sum}_{b=1}^{10}{p}_{H,b}\mathit{\log}\left({p}_{H,b}\right), $$

where p_H,b_ is the relative frequency of hexamer motif (k-mer) H occurring in bin b.

For each hexamer, 10,000 random uniform distributions with the same number of occurrences were simulated and the entropy for each distribution was calculated. Since uniform distributions have the largest possible entropy (over a finite interval), non-uniform distributions should be significantly smaller. By comparing the entropy of the actual hexamer entropy relative to the random entropy, an empirical *p*-value was calculated.

As a second criterion, to be considered a candidate hexamer motif, the distribution of hexamers was required to be significantly different in first introns compared to the distribution in other introns. A Fisher’s exact test on the binned data was used to determine whether there was a significant difference between the two distributions.

For both metrics, the Benjamini–Hochberg method of False Discovery Rate (FDR) adjustment was applied [29].

### Multiple sequence alignments/ consensus motif generation

For the identification of a consensus motif from candidate hexamers, a Multiple Sequence Alignment (MSA) on a subset of hexamers considered candidate motifs was performed. The multiple alignment using fast Fourier transform (MAFFT) tool [30] was used to perform the alignment. JalView [31] was utilized for tree visualization. For comparison of consensus motifs, the STAMP tool [32] was used. Collapse of hexamer motifs into consensus motifs is, by its nature, to some degree arbitrary and was performed requiring a minimum support per consensus position of two individual motifs and guided by the dendrogram of sequence-distance-clustered motifs (Fig. 4a) with the objective to group similar motifs together, while unique motifs should remain separate.

### Calculation of IMEter score

IMEter [20] is a tool scoring the similarity of a sequence to introns close to the TSS. IMEter version 2.2 was downloaded from the KorfLab/IME github repository. IMEter was trained with the Phytozome dataset as described in the IMEter use manual [33]. The IMEter score for each first intron was then calculated. Introns were subsequently ranked by their IMEter score.

### Detection of correlated gene expression

For detecting correlated gene expression, microarray expression data from Craigon et al. (2004) was used, covering 20,922 genes with unique probe-geneID mappings, profiled in 5295 hybridizations/ conditions [34]. The data was normalized as described in Korkuc et al. (2014) [25]. For comparing the gene expression of sets of genes, Pearson correlation of normalized, log-transformed expression levels across all samples was used. For each gene subset, the correlations between all possible combinations of two genes was calculated based on the determined expression levels in the samples contained in the expression dataset. To compare two subsets, a Cohen’s d analysis of effect size on the two sets of correlations was performed. This yielded both an evaluation of the direction as well as the magnitude of the effect. Confining the analysis to genes with introns, annotated 5’UTR with length > 0 bp, and requiring a log (median_expression) > 0.1 left 13,504 genes for expression analysis. Here, we follow the same rationale of testing for functional relevance of motifs with regard to gene expression as described in [35], where the approach is also illustrated schematically.

In general, gene subsets can be compared to a set of random genes of equal set size, or other gene subsets. To avoid correlation related to homology present within a gene subset containing a certain hexamer, comparisons to subsets of genes containing other, but specific hexamers were performed. For this, hexamers with occurrences similar to the hexamers of interest (+/− 10%) were chosen, and correlations for their respective gene subsets were calculated. Then, Cohen’s d values for the gene set containing the hexamer of interest and each of the new subsets were calculated. Finally, the mean effect size was determined.

Potential motifs were compared to high IME-scoring introns as judged by the IMEter tool. The correlation of the hexamer gene set was compared to the set of genes with the highest IMEter score with equal set size by calculating Cohen’s d effect size.

### Analysis of differentially methylated regions

For the analysis of differential methylation, information on differentially methylated regions (DMRs) from Kawakatsu et al. (2016) [24] was used. These cover three different types of methylation, CG-DMRs, representing differential methylation only in the CG context; CH-DMRs, which cover only regions that are differentially methylated in the CHG/CHH context; and C-DMRs, which are regions with differential methylation in both contexts. For all sets, all differentially methylated positions (positions that are part of DMRs) within first introns were extracted and summarized for each intron, respectively.

### Identification of new motifs and motif binding comparison

The tool Tomtom was used to compare candidate motifs to a set of 872 sequence motifs reported as part of the published DAP-seq motif dataset for *Arabidopsis thaliana* [36]. DAP-seq motifs correspond to transcription factor binding sites motifs derived from binding assays of transcription factors to “naked” genome DNA segments.

### Using natural variants to assess the effect of mutations in candidate motifs on gene expression level

For every candidate motif as detected in the reference genome sequence, all genes were identified harboring that motif in their first intron. Then, based on SNP information, for every such gene, *Arabidopsis thaliana* accessions with available expression information were divided into two sets: one containing the identified original motif in a given gene and its intron, and one with at least one mutation in the motif locus in that gene (allelic variant). The expression levels of variants without mutation were compared to the variants with mutations. Expression levels were taken as obtained from a log-transformed (natural log) upper-quartile normalized RNA-seq transcriptome dataset containing 728 accessions [24], and requiring the median expression level to be greater than one across all samples to exclude genes expressed at very low levels, where proper sample normalization is less robust. Two-sample t-tests were applied to filter for significantly different expression of the gene harboring the unmutated vs. mutated motif variant and Cohen’s d effect sizes were calculated. This was done across all genes containing the motif of interest and with identified motif-based allelic variants yielding a distribution of Cohen’s d values. This process was repeated for all identified candidate intron motifs as well as for all other (non-candidate) hexamer motifs to serve as a control.

### GO-term enrichment

Gene Ontology (GO)-term enrichment analysis was performed based on a Fisher’s exact test with FDR correction. The terms were extracted from the GO-slim-term subset available from TAIR10 [26].

### Prediction of expression level with Random Forest models

#### Selected features

All features chosen to characterize introns were directly or indirectly linked to information contained in first introns. Table 1 lists all features along with a short description. The length of the first intron, the distance of the first intron to the coding sequence, the distance of the first intron to the transcription start site and intron retainment of the proximal intron were derived from the extracted intron GFF3 file. The relative base-type frequencies were derived from the extracted FASTA file of the first introns, with the flanking three bp bordering the splice sites masked. The relative dimer counts were calculated in a similar fashion as the hexamers described above, but with k = 2. All possible dimers were determined, their occurrence in each first intron, excluding the splice sites, were assessed, and the count of reverse complementary dimers were combined. Finally, the counts were normalized by dividing by the respective intron length.

**Table 1:** Features used for the prediction of expression level based on Random Forest models

Feature	Abbreviation	Description
intron length	length	length of the first intron
distance to CDS-start	distance_CDS	distance of the first intron to the translation start codon of its gene
distance to TSS	distance_TSS	distance of the first intron to the transcription start site
IMEter score	imeter	calculated IMEter score of the first intron
SNP ber bp	SNP_per_bp	SNP rate per base pair
DMRs C context	DMR_C	number of differentially methylated areas with CG/CHG/CHH context in the intron
DMRs CG context	DMR_CG	number of differentially methylated areas with CG context in the intron
transposable elements	n_transposons	normalized number of transposable elements in the proximal intron
intron retainment	IR	“1” if first intron is retained in some isoforms as reported in the GFF file, otherwise “0”
CNS	CNS	number of conserved non-coding sequence (CNS) sections in the intron
minimum folding energy	min_fold_energy	normalized minimum folding energy of the first intron
A/T/C/G content	A/T/C/G	base-type occurrence percentage of A/T/C/G of first introns, excluding the splice sites
dimer percentages	TA/CG...	relative frequency of all possible dimers in the first intron, with reverse complement dimers combined. Splice sites are excluded

Information about differentially methylated regions (DMRs) was derived as described above. Similarly, the IMEter score for the first introns was calculated as described above. The SNP-frequency per bp was calculated using the VCF file.

The minimum folding energy was calculated using mfold [37]. For each first intron, an overhang of 20 bp into the flanking exons on both sides were included in the calculation. The minimum energy was then normalized by dividing by intron length with 40 bp for the overhang added.

For considering the presence of conserved non-coding sequences (CNS), a dataset from Haudry et al. (2013) was used [38]. A position was considered conserved if an associated CNS sequence was found present in at least four of the nine Brassicaceae species examined in [38]. The relevant positions, i.e. positions that overlapped with first introns, were extracted. For every intron, the total number of CNS positions was determined, and normalized by intron length.

Transposable elements were extracted from the TAIR10 transposable element dataset [26]. The total number of transposable elements per intron was normalized by intron length.

As an indication of functional relevance, we probed introns for evidence of retention in annotated splice variants as reported in the GFF-file. If an intron sequence was found to overlap with an exon of an alternative transcript, it was considered retainable (retention = 1), otherwise not (retention = 0).

#### Classification

As a target variable for prediction, gene expression level as reported by the above-mentioned microarray data [34] was utilized. The median expression for each gene across all samples was determined. A binary classification into high/low expression was chosen using the median as a set division threshold. To potentially increase prediction performance, models were also created for a modified dataset, which contained only genes found in the upper and lower quartile of RNA expression levels. The goal was to create two more distinct groups to allow better classification (increased contrast).

#### Model selection

For creating the actual prediction model, the Random Forest (RF) classifier as implemented in the sklearn [39] module was used. Hyperparameter tuning via random grid search with cross-validation to increase performance and reduce overfitting of the model was performed. The final RF-models contained 6000 trees. Each tree had a maximum depth of 10 with a minimum number of samples per split of 5, and a minimum of two samples at the leaf nodes. Number of features to choose from at every split was set to sqrt(total_number_of_features).

#### Dataset selection

For training the Random Forest model, the dataset for the introns was randomly split into training and test dataset with a ratio of 80 and 20%. For the ROC curve analysis, ten-fold cross-validation on the whole set was performed.

#### Feature importance

For determining the feature importance, permutation feature importance was selected. It has been suggested that this method provides better results than the “Mean Decrease in Gini” method, which is used by the sklearn classifier [40]. After training the classifier, one feature of the test set was permuted randomly and the accuracy was scored. This was repeated five times for each feature, and the mean decrease in accuracy (MDA) was calculated, respectively. This process was repeated for all features.

#### SHAP importance

The Shapley Additive explanation (SHAP) method explains individual predictions of a model [41]. It is based on Shapley Values, which have their origin in game theory. A Shapley value of a feature is the average contribution to all possible feature combinations. Calculation of Shapley values is computational expensive due to combinatorial explosion. SHAP therefore uses sampling to approximate Shapley values to reduce the computational burden. The Python package SHAP [42] was used to calculate SHAP values for the trained models, and to visualize the results.

### Statistical analysis and visualization

All statistical analyses were done in Python 3.7 [43]. The modules scipy [44], numpy [45], and pandas [46] were used. Visualization and plotting was performed with the modules matplotlib [47] and seaborn [48]. In cases of single test statistics, reported *p*-values less than *p* = 0.001 are not specified further (precision) and indicated as *p* < 0.001.

### Code availability and additional set data

Code and scripts developed and used in this study are available at https://github.com/georgback/IME or via 10.5281/zenodo.4749386. For the five reported consensus and the two IMEter motifs, associated lists of genes harboring them in their first intron are made available as a Supplementary data file.

## Results

**Fig. 1:**
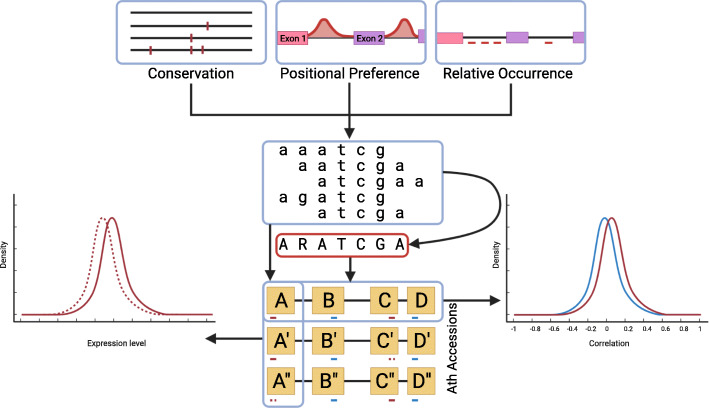
Schematic workflow. Based on conservation across Arabidopsis accessions containing SNPs (vertical red bars), positional preferences (indicated as frequency profiles), and occurrence differences of hexamers in first introns relative to other introns (horizontal bars illustrate a particular candidate hexamer), candidate hexamer motifs were identified. To test for functional relevance, correlation of gene expression among genes containing a potential motif was compared to correlations of gene expression of sets of genes containing hexamers with comparable frequency. Hexamers with the highest correlation were selected and consensus motifs were determined. To validate both hexamer and consensus motifs, natural variations among *Arabidopsis thaliana* accessions were utilized. For genes containing a motif of interest in their first intron and with detected naturally occurring mutations, accessions were split into the canonical/reference (containing the original motif) and the non-canonical/variant (mutated motif) allele set, and expression levels of the different alleles were compared. Figure created with BioRender.com

The primary objective of this study was to identify novel IME-inducing intron motifs. In the following, we shall describe the rationale and workflow for their identification and functional characterization. To support this verbal description, Fig. 1 provides a schematic graphical illustration.

### Comparison of SNP-frequencies in first versus other introns

**Fig. 2:**
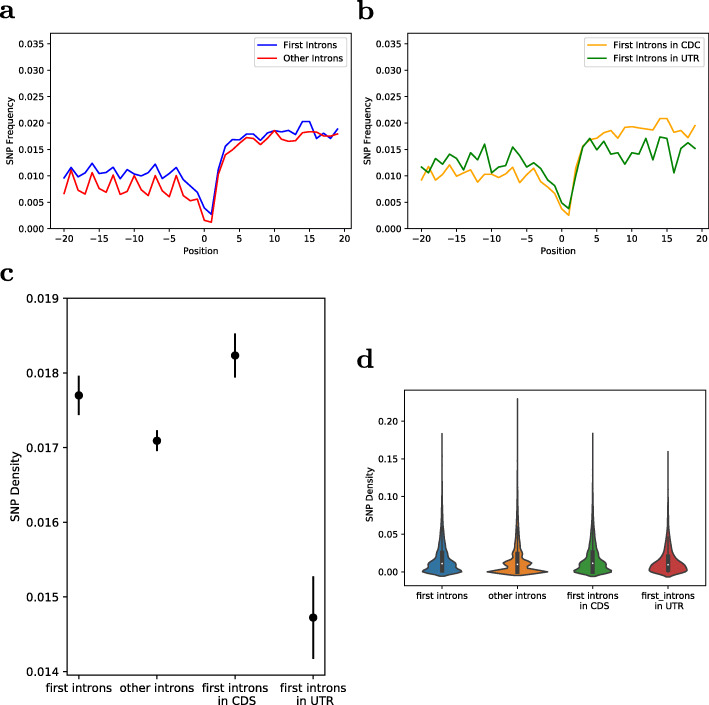
Comparison of SNP-frequencies of intron subsets. (**a**) Average relative SNP-frequency of the first 20 bp of the first introns compared to the other introns including the last 20 bp of the preceding exons (**b**) Average relative SNP-frequency of the first 20 bp of first introns in 5′-UTRs compared to first introns in CDS including the last 20 bp of the preceding exons (**c**) Comparison the average SNP-frequency per bp (SNP-density) and confidence intervals of different intron subsets (**d**) Violin plots of SNP-frequencies per bp (SNP-densities) of different intron subsets. In (**a**) and (**b**) positions are relative to the exons-intron junction with zero denoting the first intron position

Since it has been shown that specifically the first intron bears the capacity to influence expression of the gene it is part of, the set of Arabidopsis introns was split into two sets, one with only the first introns, i.e. the 5′-most, of each gene, and another for all remaining introns, termed “other introns”. The average intron length of first introns was determined as 259.7 bp, with a median of 161 bp, and a mean of 160.8 bp for the other introns, with a median of 100 bp, respectively. For both intron sets, the respective SNP-density was calculated by using the variants data of the 1001 Arabidopsis genome project [23]. Only positions with at least 50 alleles containing a different variant (minor allele) were considered as SNP positions, and the first and last three positions of each intron were excluded to avoid over-representation of splice sites. Surprisingly, first introns were observed to have a slightly higher SNP-density of 0.0164 SNPs (i.e. polymorphic positions) per bp compared to the other introns with 0.016 SNPs per base position. These mean values reflect the global average. The associated averages per intron are 0.177 and 0.171, respectively (Mann–Whitney U test, *p* < 0.001, distributions shown in Fig. 2). A visualization of the relative SNP-frequency for the first (5′ end of intron) 20 bp positions, including a 20 bp overlap into the preceding exon clearly shows this difference (Fig. 2a). This effect is not only observable in the introns itself, but also in the preceding exons, likely explained by the embedding of other introns in coding regions with associated conservation pressure, whereas first introns are often found in a non-coding UTR context. The position-resolved conservation profiles (Figs. 2a, b) also confirm the expected lower SNP-frequency on and near the exon/intron splice site as well as the expected three-bp periodicity within the exon/coding region. To test whether the difference in conservation effect is related to the positioning of introns in the 5′ untranslated region (UTR), which could potentially explain reduced conservation, first introns were separated into introns positioned in the 5′-UTR and introns positioned in the CDS. Surprisingly, first introns in 5′-UTRs were found to have a lower SNP-density than first introns in the CDS, with an average SNP-density per intron of 0.0147 for the 5′-UTR introns and 0.0182 for the CDS introns (Mann–Whitney U test, *p* < 0.001) (Figs. 2b, c, d). By contrast, upstream intron-flanking regions showed the expected behavior with UTR-exons being less conserved than CDS-exons (Fig. 2b).

High sequence conservation, as reflected by a low SNP-density, can be an indicator of functionality [49]. This agrees well with IME-function predominantly being found in introns close to the TSS and therefore close to (or even within) the 5′-UTR, indicating a possible correlation between conservation and IME function, but within CDS regions, first and other introns do not follow the expected conservation pattern.

### Selection criteria for potential cis-regulatory intron motifs

For identifying candidate intron motifs associated with IME, a k-mer-based strategy similar to IMEter was applied, with additionally utilizing conservation and relative position in introns as informative criteria, similarly as described by Korkuc et al. (2014) [25]. As a compromise between specificity of a sequence motif and combinatorial explosion, a k-mer length of k = 6 was chosen. All counts of reverse-complement hexamers were combined, leading to a total of 2080 unique potential 6-mer (hexamer) motifs. Four properties were examined for determining whether a hexamer was considered a candidate: 1) higher sequence conservation in first introns than in other introns, 2) higher relative occurrence in first introns than in other introns, 3) non-uniform distribution of the motif within the first intron, and 4) dissimilar positional distribution of the motif between first and other introns. Criteria 3 and 4, which impose positional preferences, were introduced to follow the rationale that similarly to transcription factor binding sites [25, 50], intronic motifs may exhibit such positional preferences as well. Of those criteria, criterion 2 follows the approach of IMEter, while criteria 1, 3, and 4 are introduced in addition in this study.

#### Evolutionary conservation of hexamers

**Fig. 3:**
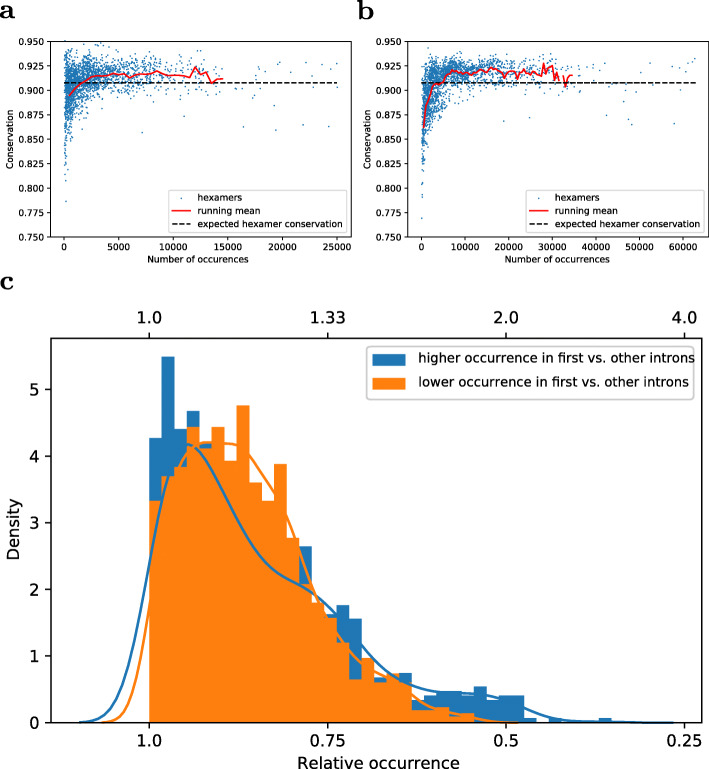
Hexamer characteristics. Conservation and occurrence of hexamers in (**a**) first introns, (**b**) other introns, **(c)** Comparison of hexamers relative occurrence distributions of hexamers that occur more (blue, top x-axis)/ less (orange, bottom x-axis) often in first than in other introns. In (**a**) and (**b**), for definition of conservation, see Methods. Every dot represents a hexamer, the red line represents a computed running average, and the dashed black line corresponds to the respective estimated random conservation based on Eq. 2

Our approach builds on the rationale that functional motifs show increased conservation. Therefore, and if indeed IME is associated specifically with first introns, we expect potential motifs to be more evolutionarily conserved in first introns than in other introns. The mean conservation rate (see Methods for definition) over all hexamers was determined as 0.9131, higher than the randomly expected rate, C_r_, Eq. 2, of 0.905 (Fig. 3a). Similarly, other introns had an average hexamer conservation of 0.915 compared to the expected value of 0.907 (Fig. 3b). At first, it may seem surprising that the average observed hexamer conservation is higher than that based on the expected background conservation (Eq. 3). This apparent contradiction can be explained as an indication that SNPs are not completely randomly distributed within introns, but tend to positionally cluster. Similar observations have previously been reported [51]. This could be due to either a bias in the sequencing technology or some biological reason. Also, hexamers with very low occurrences tend to have higher SNP-rates (Figs. 3a, b). This may point to a sequencing artifact as well (homo-oligomeric stretches). A total of 929 hexamers were determined to have a higher conservation in first introns relative to other introns, while 1151 hexamers were more conserved in other introns, which reflects the observed higher SNP frequency, and hence, lower conservation, in first vs. other introns (Fig. 3a).

#### Relative occurrence of hexamers in first vs. other introns

Under the assumption that functional sequence motifs induce IME, it appears plausible to expect that these motifs show a higher relative occurrence in first introns compared to other introns, since the vast majority of reported IME-introns are first introns of a gene [20]. Inspecting relative hexamer counts (count of a particular hexamer divided by the total number of detected hexamers), 843 hexamers were detected with higher relative occurrence in first compared to other introns, while for 1237 hexamers, the inverse was true. A closer examination of the relative count distribution of hexamers revealed a significant difference between the distribution of hexamers with lower relative frequency versus those with higher relative frequency in first introns (Fig. 3c, Kolmogorov-Smirnov test *p* < 0.001). While there are fewer hexamers with higher relative occurrence in first vs. other introns than what is observed in reverse, those that are overrepresented in first introns show a pronounced tail (at around a twofold enrichment factor) that may point to the ones that are functionally significant and, thus, enriched.

#### Non-uniform positional distribution of hexamers in introns

Studies have shown that functional sequence motifs often exhibit a positional preference [25, 50], including signals associated with IME [20]. Assuming that potential functional motifs in introns exhibit this preference as well, hexamer positional distributions were tested for deviation from uniformity (see Methods), yielding 1448 hexamers detected with significantly non-uniform positional distributions in first introns.

To exclude positional preferences unrelated to hexamer IME function, only hexamers with significantly different positional preferences in first and other introns were considered further. A Fisher’s Exact test comparing positionally binned distribution of hexamers (ten bins, see Methods) within first introns to other introns respectively yielded a subset of 459 hexamers, which were significantly differently distributed in first vs. other introns.

In total, 81 hexamers met all four requirements laid out above, and were investigated further.

### Analysis of identified candidate hexamers

#### Expression correlation of genes containing candidate intronic hexamer motifs

To test for any regulatory effects of the identified 81 candidate first-intron motifs, at first, correlation of gene expression level was taken as an indicator, while later, we also inspected expression level. Under the assumption that an intron motif regulates gene expression, those genes that harbor a particular motif should exhibit a higher correlation of gene expression amongst them than a comparable set of random genes. However, increased correlation among genes with a specific intron motif could not only indicate regulatory effects, but also originate from the genes being homologous. Closely related genes might exhibit a similar expression profile and will also be more sequence-similar to one another with a correspondingly increased probability to find the same hexamer in their introns. Therefore, candidate motifs were compared to hexamers with similar occurrences as the one under consideration (within a 10% interval of higher/lower occurrence) to account for this effect. Gene expression correlation of the gene subset containing the hexamer of interest was computed, and then compared to the correlation of genes observed to each contain a comparable hexamer in their first intron. Of note, as a control, we compared the matching k-mer approach to the naive approach to simply use all other genes and found concordant results (Supplementary Fig. S1).

The median Cohen’s d effect size, i.e. the magnitude of the difference of correlation values for the two gene sets across all 81 motifs was 0.018 (std.dev. = 0.029), with only 10 hexamers having a negative mean effect size (Table 2; for the complete set of 81 candidate motifs, see Supplementary Table 1). Thus, a significant majority (71 in total) of the 81 selected hexamers exhibited higher correlation than hexamers of similar occurrence (*p* = 1.8E-12, binomial test, with p_prior_ = 0.5). Sixteen candidate motifs with a mean effect size of greater than an arbitrarily chosen threshold of + 0.05 (5%) were selected and investigated further.

**Table 2:** Hexamers with potential regulatory function as evidenced by increased conservation, positional preferences, and co-expression of genes harboring respective motifs in their first introns. ‘Cohen’s d correlation’ is the effect size of difference in the distribution of correlation coefficients between the expression levels of genes harboring the respective motif relative to a gene set containing frequency-matched random hexamer motifs across all experimental conditions present in the expression dataset. ‘Cohen’s d expression level’ refers to the effect size related to expression level of genes containing the respective motif in the first intron relative to all other intron-harboring genes. Listed also are the numbers of genes, in which the respective intron motif was found. Listed are all motifs with ‘Cohen’s d expression’>0.05. For a complete listing of all 81 candidate motifs, identified based on conservation and positional preference alone, see Supplementary Table 1

Hexamer	Cohen’s d, Correlation, comparable, random hexamer	Cohen’s d, Expression level	Number of genes
AGATCG	1.45E-01	0.46	1807
ACCCTA	9.82E-02	0.18	2964
TCGATC	9.16E-02	0.34	2014
TCGGAG	8.58E-02	0.27	857
TCTCGC	8.13E-02	0.19	785
GATTCG	7.68E-02	0.32	2516
ATCGAA	7.07E-02	0.31	4188
AAATCG	7.00E-02	0.28	4086
AATCGA	6.88E-02	0.31	4406
TTAGGG	6.76E-02	0.19	2896
ATCGAG	6.20E-02	0.28	1773
TCTCGA	5.79E-02	0.22	2044
CTCTCG	5.77E-02	0.23	1124
AAACCC	5.33E-02	0.18	4970
TTCTCG	5.27E-02	0.19	2188
TTTCGA	5.20E-02	0.21	3866

#### Analysis of candidate hexamer subset with evidence of expression regulation

For each of those 16 hexamers, the average gene expression level of genes harboring them in their first introns was significantly higher than the average of the whole set (*p* < 0.001), with Cohen’s d effect size ranging from 0.18 for ACCCTA to 0.45 for AGATCG. This result is in line with motif-mediated IME being associated with highly expressed genes.

### Candidate consensus motifs and gene function association

**Fig. 4:**
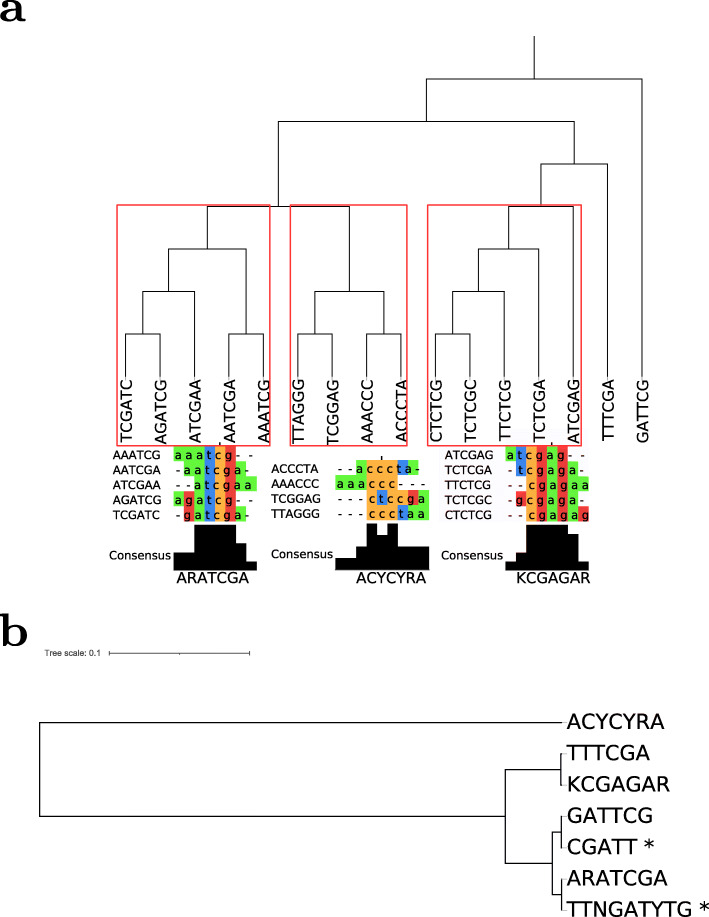
Consensus motifs and comparison to IMEter motifs. **a** Consensus motifs generated by Neighbor Joining Tree of MAFFT alignments of the 16 candidate hexamers with clusters indicated as boxes. The clustering threshold for collapsing motifs into consensus motifs, shown below to the respectively clustered motifs, was set based on visual inspection. Consensus motifs were required to be supported by two or more bases, i.e. vertically aligned motif positions. **b** Sequence comparison of the five consensus motifs and the two previously reported IMEter motifs (indicated by *). Dendrogram was created using the tool Stamp. Branch lengths are proportional to distance

The set of hexamers contained sequence-related hexamer sequences, which may be equivalent in function or part of a larger consensus motif, e.g. AGATCG and TCGATC (with its reverse-complement GATCGA). Using the program MAFFT, the set of 16 motifs was collapsed into five motifs, GATTCG, TTTCGA, KCGAGAR, ACYCYR, and ARATCGA. Three of these are consensus motifs from several individual hexamers, and two remain as their original hexamer definition (Fig. 4a).

Next we tested whether the identified motifs correspond to known binding sites of known DNA-binding proteins, such as transcription factors. A motif comparison analysis performed with the motif comparison tool Tomtom against the DAP-seq database for Arabidopsis transcription factors and their associated target motifs revealed no significant overlap (all E-values> 1) with any of the 872 DAPseq-reported motifs.

To elucidate the biological role of the genes harboring the candidate motifs, a GO-term enrichment analysis for genes, whose first introns contain the five motifs were performed, with gene sets considered separately for every motif (Table 3). With regard to GO-cellular components, gene sets for all motifs were significantly enriched for cytosol and cytoplasmic components, with gene sets associated with three motifs being significantly enriched for Golgi apparatus (ARATCGA, GATTCG, TTTCGA), and two for endoplasmatic reticulum (ARATCGA, GATTCG) and nucleus (ACYCYRA, KCGAGAR), respectively. All motif sets were significantly depleted for mitochondrial and extracellular genes, with four sets being also depleted for chloroplast genes (ACYCYRA, ARATCGA, GATTCG, KCGAGAR). Testing GO-function terms, all motifs were found enriched for protein binding. Furthermore, structural molecule activity (ARATCGA, GATTCG, TTTCGA) and DNA/RNA-binding (ACYCYRA, ARATCGA, GATTCG) were overrepresented in gene sets of three motifs, respectively. The GO-term “unknown molecular function” was significantly underrepresented for all motifs. Additionally, gene sets of three motifs were depleted for transcription factors (ARATCGA, GATTCG, TTTCGA). Lastly, significantly enriched process terms were and DNA/RNA metabolism (ACYCYRA, ARATCGA, KCGAGAR, TTTCGA), cell organization (ACYCYRA, ARATCGA, KCGAGAR, TTTCGA), and transport (ARATCGA, GATTCG, KCGAGAR, TTTCGA) with four motif sets each, while signal transduction (ARATCGA, GATTCG, KCGAGAR, TTTCGA) and unknown processes (all) were underrepresented. Thus, generic housekeeping functions appear overrepresented, while signaling and transcription factor activities appear to be less present in the gene sets associated with the five identified IME-candidate-motifs.

**Table 3:** Enriched and depleted GO-terms in gene sets harboring the identified consensus intron motifs in their first introns. For every GO-term, significant FDR-corrected *p*-values (p_FDR_< 0.05) based on Fisher exact tests are listed (correction per GO-category) or left empty if p_FDR_>0.05

GO- category	GO-term	ACYCYRA	ARATCGA	GATTCG	TTTCGA	KCGAGAR
**component**	*enriched*					
	cytosol	1.16E-06	2.08E-07	6.42E-05	3.66E-03	5.21E-05
	Golgi apparatus		6.10E-07	1.61E-04	3.72E-02	
	endoplasmatic reticulum		5.56E-03	2.41E-02		
	nucleus	2.78E-12				2.49E-02
	*depleted*					
	mitochondria	8.15E-04	1.04E-02	2.41E-02	4.17E-02	2.49E-02
	extracellular	1.84E-12	1.11E-14	5.44E-14	8.27E-13	5.92E-11
	chloroplast	4.12E-02	6.09E-04	1.34E-02		3.16E-02
**function**	*enriched*					
	protein binding	7.47E-11	3.69E-07	1.12E-04	1.81E-05	1.19E-4
	structural molecule activity		1.36E-03	1.56E-02	3.44E-04	
	DNA or RNA binding	8.06E-05	8.31E-03	2.64E-02		
	*depleted*					
	unknown molecular function	7.47E-11	2.45E-09	4.30E-04	7.35E-11	2.81E-05
	transcription factor activity		1.68E-03	2.64E-02	1.54E-02	
**process**	*enriched*					
	DNA or RNA metabolism	3.65E-06	1.82E-04		6.25E-04	8.49E-04
	cell organisation	4.84E-03	3.27E-04		3.64E-02	1.29E-02
	transport		3.39E-03	1.79E-02	6.21E-04	4.78E-02
	*depleted*					
	unknown processes	6.93E-11	7.53E-10	2.07E-03	1.24E-06	8.49E-04
	signal transduction		1.13E-02	1.79E-02	7.91E-03	1.53E-02

### Comparison of potential regulatory motifs to IMEter

**Fig. 5:**
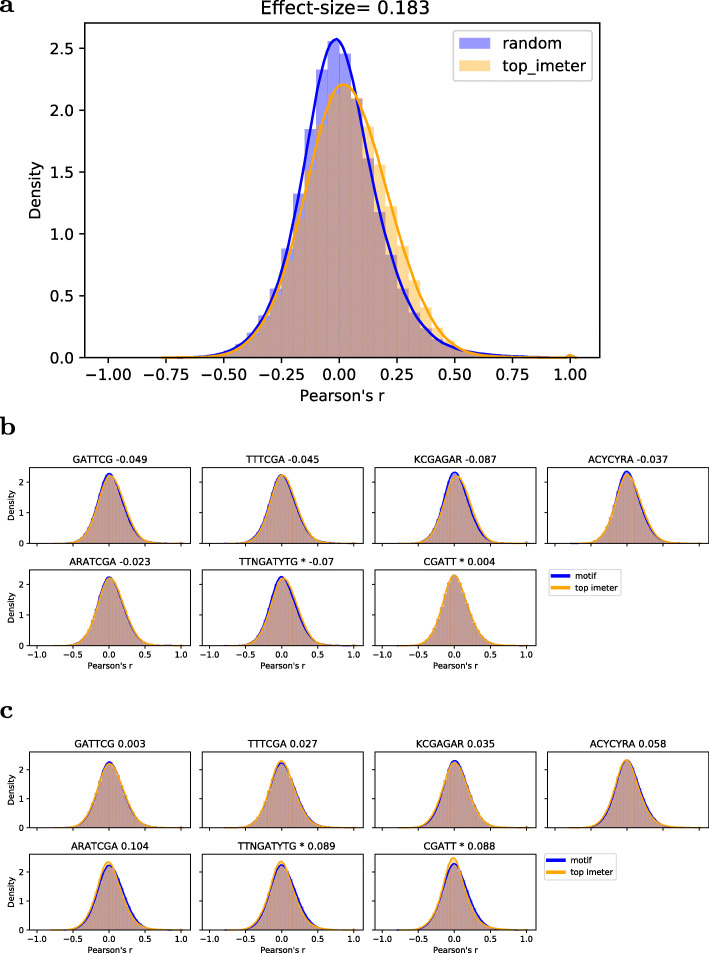
Comparison of the five identified consensus motifs to IMEter. Density plot of intra-set Pearson correlation of gene expression comparing (**a**), the 2000 genes with the highest IMEter score (top_imeter) to a subset of random genes of equal size, (**b**), genes containing of one of the five identified candidate motifs and genes containing the IMEter-derived motifs TTNGATYTG and CGATT, designated by asterisks. Each gene set was compared to a subset of genes with the highest IMEter score of equal set size and their difference expressed as Cohen’s d effect size, given in the graph titles, with negative values suggesting smaller effect in motif-set relative to size-matched IMEter-score set. (**c**) same as in (**b**), but with overlapping genes (genes found in both sets) removed from the IMEter set. Intra-set Pearson correlation refers to the determined Pearson correlation coefficients of all possible gene pairs in sets of genes harboring the same motif or belonging to the same top-IMEter set

To further evaluate the newly identified motifs, they were compared to IMEter, the most commonly used tool for identifying potentially IME introns. IMEter scores whole introns [21], or, in a new version, a sliding window of 50 bp [20]. For all first introns, the IMEter score was calculated, and then sorted by score. Genes with the highest scoring introns were correlated amongst themselves at significantly higher levels than a subset of random genes of equal set size (*p* < 0.001), with an average Cohen’s d of 0.183 (Fig. 5a). The mean expression level of the top 2000 IMEter score genes was significantly higher than that of the whole gene set (*p <* 0.001, Cohen’s d effect size of 0.43). By comparison, correlation of expression amongst genes containing either one of the five consensus motifs reported in this study was either comparable to or only slightly below that of the IMEter set (Fig. 5b, |Cohen’s d| < 0.1), suggesting a potentially cis-regulatory role.

The overlap between the candidate motif gene sets and the corresponding IMEter sets of equal size was large, with an average overlap of 34% (*p <* 0.001, regarding the overlap in percent, note that sets were always of the same size). This is expected, since both approaches partly employ similar strategies for identifying IME. When the candidate motifs were compared to the IMEter set not containing overlapping genes, the effect size associated with the candidate motif gene set generally increased, resulting in comparable gene expression correlation of genes within the respective gene sets as observed for the size-matched top-IMEter gene sets, with effect size ranging from 0 to 0.1 (Fig. 5c), suggesting an even stronger, albeit slightly, regulatory effect associated with the identified five consensus motifs compared to IMEter-selected gene sets.

Based on the IMEter tool, Rose et al. (2008) [21] and Parra et al., [20] identified two motifs, CGATT and TTNGATYTG, which were overrepresented in introns with high IMEter scores, and were shown to be associated with induction of gene expression [52]. Judged by their sequence, of the five identified consensus motifs two motifs (ARATCGA, GATTCG) show some resemblance with the two IMEter motifs, albeit not identical, while three motifs (ACYCYRA, TTTCGA, KCGAGAR) can be considered more distinct, and thus, potentially novel functional IME motifs (Fig. 4b).

Compared to the top-scoring IMEter genes, genes containing either one of the two IMEter motifs had a comparable correlation amongst each other, with associated Cohen’s d of 0.004 and −0.072, respectively (Fig. 5b, asterisk-labeled motifs). With overlapping genes removed from the IMEter set, Cohen’s d increased to 0.088 and 0.089 (Fig. 5c, asterisk-labeled motifs), thus, requiring the motifs to be present alone yielded a significant co-expression signal. The two IMEter motifs exhibited a significantly higher mean expression than the total set (*p* < 0.001), with effect size of 0.31 for CGATT and 0.37 for TTNGATYTG. By comparison, our consensus motifs were found with corresponding effect sizes of ARATCGA: 0.33, GATTCG: 0.32, KCGAGAR: 0.23, TTTCGA: 0.21, ACYCYRA: 0.14 (mean: 0.25). Thus, the two consensus motifs detected as sequence-similar to the reported IMEter motifs (ARATCGA and GATTCG) showed the largest effect size and comparable to the two IMEter motifs, while the other three consensus motifs were found with slightly lower, but still very strong effects.

Taken together, our consensus motifs resulted in similar effects as compared to the IMEter-based intron scoring and the two IMEter-motifs, and yielded novel motif definitions and/or altogether novel motifs that may function in a cis-regulatory fashion.

### Test for effect of naturally occurring mutations in candidate motifs on gene expression

With the availability of both sequence and expression information for many Arabidopsis accessions under controlled and identical conditions, it is possible to probe for the effect of mutations in candidate motifs on gene expression, allowing to test the hypothesis that mutated motifs cause lowered gene expression relative to levels of allelic versions of that gene containing the unmutated original motif version as found in the reference genome.

**Fig. 6:**
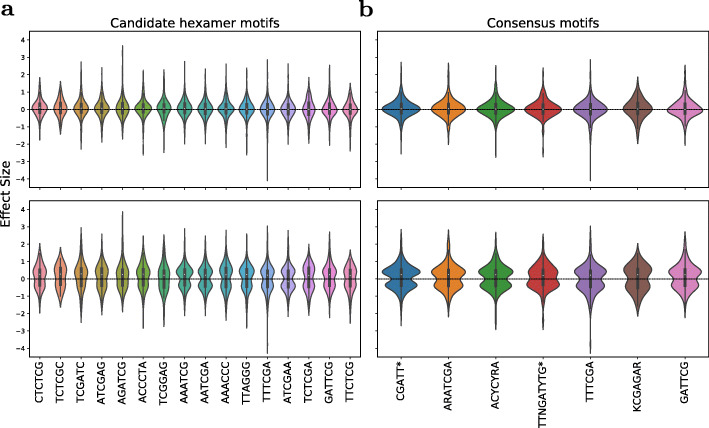
Effect of mutations in candidate motifs on gene expression level. For every candidate motif ((**a**) hexamers; (**b**) consensus motifs) the difference of gene expression of allelic variants of every gene containing it was compared. Allelic variants as present in different Arabidopsis accessions were grouped into two sets of variants: 1) motif present as defined, 2) motif locus found with at least one mutation. Distributions show effect sizes (Cohen’s d) of differences across all genes with two such allele-groups as present in the different *Arabidopsis thaliana* accessions based on available RNA-seq expression data. Upper row shows all effect sizes, lower row only for those gene, for which the allelic expression difference was significant (t-test, *p*-value< 0.05 without correction for multiple testing as the goal was not to identify true-positives, but to apply a filter that imposes some level of significance of difference). For further summary statistics, see Table 4

Indeed, for most of the 16 hexamer motifs and the seven candidate consensus motifs (five identified in this study, two IMEter motifs), expression levels of allelic variants harboring the unmutated motif were found with increased expression relative to versions of those genes (i.e. present in different accessions = allelic variants) with the motif locus containing mutations (Fig. 6, Table 4). Based on median effect sizes, 14 of the 16 hexamer motifs were positive (Table 4, column B) with associated Fisher exact test *p*-values of *p* = 0.029, so were 6 out of the 7 consensus motifs, though significance could not be established for the latter due to the small number of only seven motifs (*p* = 0.19). In the Fisher exact test, all 2064 non-candidate motifs were taken as controls (1288 of which were detected with positive median Cohen’s d). Note that this reference applies to the hexamer motifs only, as for consensus motifs, their lengths vary and the collapse of motifs into a consensus is not properly reflected. Yet, for completeness, we provide the *p-*value for consensus motifs as well. The evidence for higher gene expression of unmutated- vs. mutated-motif-containing alleles was even stronger when considering only those genes with significant expression differences (up or down) between their two versions (mutated vs. unmutated, Fig. 6, bottom panels, Table 4), with 15 out of 16 motifs observed with positive median Cohen’s d (*p*_FisherExact_ = 0.015 with non-motif hexamers taken as control; Table 4, column E) as well as greater mean values (Table 4, column A (all) vs. D (significant genes), paired t-test *p-*value = 1.2E-04). Of all seven consensus motifs, six showed increased mean Cohen’s d values (*p* = 0.015). Notably, our five consensus motifs proved of very similar strength as the two IMEter motifs as judged by effect size. The increased significance for IMEter motif “CGATT” is explained by the higher occurrences due to shorter (5 bp) motif size.

**Table 4:** Test for effect of mutations in candidate motifs on gene expression. Table lists the summary statistics for the observed expression level differences of mutated vs. original motif allelic variants for all 16 individual hexamer motifs, the derived five consensus motifs, and the two IMEter motifs (designated by asterisks), and sorted by median values of Cohen’s d. Positive d-values indicated higher gene expression of allelic variants containing the original motif relative to alleles with at least one mutation in it. Associated frequency distributions are shown in Fig. 6. *P*-values refer to one-sample t-tests to test for greater than zero mean and non-parametric equivalent (Wilcoxon signed-rank test) with applied correction for multiple testing (FDR-false discovery rate). FDR-corrected *p-*values less than 0.1 (< 10% False Discovery Rate) are highlighted applying bold font. “significant genes” refers to genes with found significant expression differences between the two allelic sets (original motif vs. mutated motif) as detected by two-sample t-tests (not corrected for multiple testing as this was introduced as a filter, not as a strict selection criterion). “number of genes” refers to the number of genes with motifs found present and with two allelic variants (original and mutated). “+/−0.00” indicates zero-rounded positive/negative numbers

	A) Overall mean, Cohen’s d	B) Overall median, Cohen’s d	C) p_FDR_-value t-test, difference from zero	D) Mean Cohen’s d, significant genes	E) Median Cohen’s d, significant genes	F) Number of genes	G) Number of significant genes	H) Number of significant genes, positive Cohen’s d	I) p_FDR_-value Wilcoxon signed-rank test for significant genes
**Hexamer motifs**									
CTCTCG	0.09	0.10	**0.08**	0.14	0.27	93	49	29	0.16
TCTCGC	0.09	0.08	0.13	0.13	0.28	57	32	18	0.17
TCGATC	0.09	0.07	**0.08**	0.18	0.29	180	94	59	0.10
ATCGAG	0.07	0.06	**0.08**	0.11	0.24	162	89	51	0.15
AGATCG	0.12	0.06	**0.08**	0.18	0.25	139	80	46	0.14
ACCCTA	0.07	0.06	**0.08**	0.13	0.27	210	101	61	0.10
TCGGAG	0.04	0.06	0.31	0.04	0.21	62	34	19	0.47
AAATCG	0.07	0.05	**0.04**	0.13	0.26	360	210	125	**0.03**
AATCGA	0.04	0.03	**0.08**	0.06	0.21	411	231	120	0.17
AAACCC	0.05	0.04	**0.08**	0.09	0.24	464	233	130	0.10
TTAGGG	0.02	0.02	0.31	0.05	0.25	220	108	63	0.17
TTTCGA	−0.00	+ 0.00	0.55	−0.00	0.22	381	204	105	0.42
ATCGAA	0.02	+ 0.00	0.31	0.02	−0.16	386	203	100	0.42
TCTCGA	0.03	+ 0.00	0.31	0.04	0.19	171	104	54	0.25
GATTCG	0.06	−0.01	**0.08**	0.12	0.24	248	137	77	0.10
TTCTCG	−0.01	−0.02	0.55	−0.00	0.19	164	95	49	0.40
**Consensus motifs**									
CGATT*	0.06	0.04	**0.00**	0.11	0.25	827	441	249	**0.00**
ARATCGA	0.06	0.04	0.10	0.11	0.24	264	147	82	0.20
ACYCYRA	0.04	0.02	0.14	0.07	0.23	377	199	108	0.21
TTNGATYTG*	0.04	0.01	0.17	0.08	0.20	173	92	48	0.21
TTTCGA	+ 0.00	+ 0.00	0.51	+ 0.00	0.22	381	204	105	0.33
KCGAGAR	0.03	0.03	0.30	0.03	0.24	139	83	43	0.21
GATTCG	0.06	−0.01	0.11	0.12	0.24	248	137	77	0.21

### Effect of differential methylation in first introns on gene expression

A study of vertebrates by Anastasiadi et al. (2018) has shown a strong inverse correlation between methylation in the first intron and gene expression [12]. They also showed that first introns exhibit the highest density of differentially methylated regions (DMRs) of any genomic feature and that certain DMRs could positively correlate with gene expression. These findings suggest a potential influence of DMRs on IME and therefore on gene expression, which was further investigated here using published DMR data [24]. The gene sets associated with the two methylation contexts, CG- and C-DMRs, that each were found with sufficient numbers of observations (CH-DMRs were not considered as fewer than 100 cases of overlaps with first introns were observed) had very little overlap with either the top scoring IMEter genes, or any of the potential hexamer motif gene sets. Genes containing C-DMRs in their first intron were significantly more correlated than a set of random genes, with an average effect size of 0.1. However, C-DMR genes had a significantly lower gene expression than the set average (*p* < 0.001) with an effect size of −0.74. Conversely, genes with intronic CG-DMRs were expressed at significantly higher levels than the set average (*p <* 0.001) with an effect size of 0.07. Yet, the CG-DMR subset showed a comparatively lower expression correlation than the C-DMR set, with only 0.054 as the average effect size compared to a subset of random genes of equal set size. With regard to overlap of DMR-set genes and the gene sets associated with any of the five candidate motifs reported here, for C-DMR, no significant overlap was detected. By contrast, the CG-DMR genes overlapped significantly with all five consensus motifs (*p* < 0.004), with enrichment factors of 1.2-fold and higher.

In conclusion, no coherent picture emerges with regard to the role of DMRs in IME. While genes with CG-DMRs in their first introns are expressed at higher than average levels, the corresponding set of genes does not show correlated gene expression, a feature that we considered evidence of regulation used to identify IME motifs.

### Random Forest model for prediction of expression level based on intron features

IME has been connected to highly expressed genes such as housekeeping genes. Thus, it appears possible to cast the problem of identifying features responsible for IME as a feature extraction problem with Machine Learning methods applied to the prediction of expression level. By only including features of the first intron, the goal was to investigate the predictive value of first introns with regard to expression level of their respective genes. Random Forest classifiers were trained for the prediction of expression level. Initially, genes were binned into two groups, sets with high and low expression level, respectively, used as classes for building the classification models, with the global median expression level taken as the threshold value. To increase contrast, binning of genes was performed based on the upper and lower quartile of expression levels. A spectrum of sequence-dependent and sequence-independent intron features, which we considered potentially predictive, were selected and tested (Table 1).

**Fig. 7:**
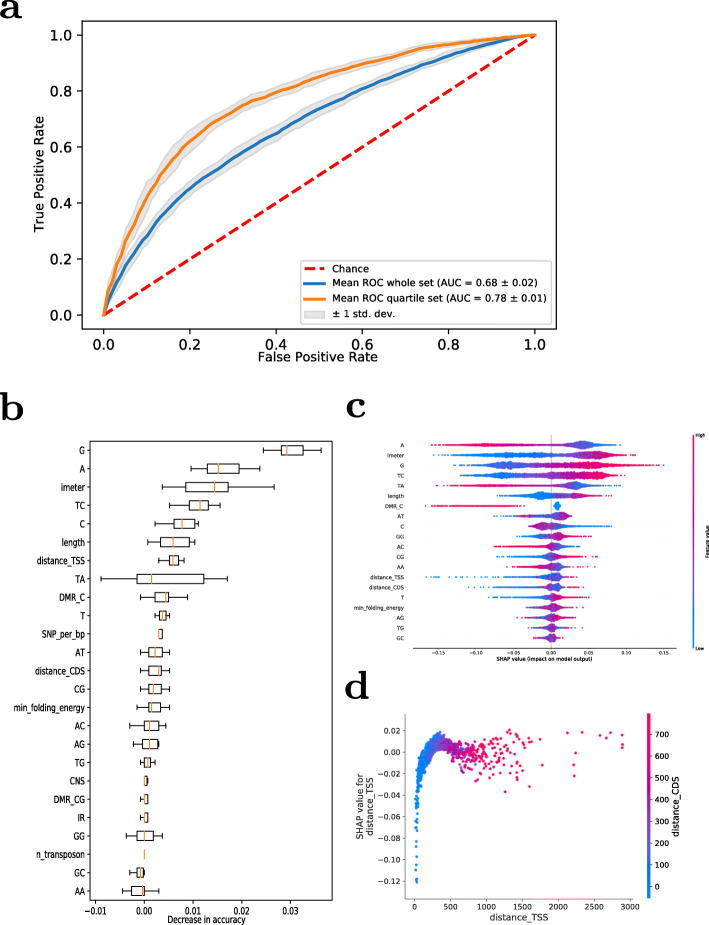
Prediction of gene expression levels based on intron features. Random Forest Performance and Feature Importance. (**a**) 10-fold Cross-validated ROC curves for Random Forests trained with the median-split (whole) set and quartile expression set, respectively, (**b**) MDA feature importance for Random Forest model trained with the lower and upper quartile expression dataset, For feature explanations, see Table 1. (**c**) SHAP summary plot of Random Forest model trained with the lower and upper quartile expression dataset, (**d**) SHAP value to feature value plot for distance to TSS, with the respective distance to CDS-start values color-coded. Positive SHAP values indicate an association with the high expression class, negative SHAP, association with the low expression class of genes

Using the median-split gene classes, the achieved model performance was modest (area under the ROC (AUC) of 0.68 and an average accuracy in a tenfold cross-validation of 63%. When increasing the gene expression difference between the two considered gene sets by using the upper and lower quartile of expressed genes to train models, a substantial increase of model performance was observed (AUC = 0.78, accuracy = 72%) (Fig. 7a).

#### Feature importance

For the trained Random Forest models, feature importances, as reflected by the mean decrease in prediction accuracy (MDA), were determined. For the best performing model, sequence composition features seemed most important, with the percentage of guanine (G) and adenine (A) having the highest impact on model performance (Fig. 7b). IMEter score, which is derived from the distribution of pentamer-motifs in introns, the relative occurrence of TC-dimers and percentage of Cytosine (C) were also found to have high feature importance, further suggesting sequence-dependent effects. Finally, intron-length and distance to the TSS had only a small positive effect on prediction performance. This is surprising, since IME has been closely associated with distance to the TSS. However, MDA, while powerful, is susceptible to correlated features, as influences can be masked.

SHAP values are an alternative and very informative way to assess feature importance and decision making of a model. They are calculated for all predictions individually, making them ideal for analyzing the effect of feature values on the prediction. For the model at hand, positive SHAP values indicate that this feature value increases the chance of the model classifying the sample as highly expressed, while negative SHAP values increase the chance of low expression classification. The importance of features determined by SHAP was assessed similarly as by MDA (Fig. 7c). Sequence features, such as A-, G-, TC-content, and IMEter-score again had the biggest impact on model prediction. Low values for A-content resulted in positive SHAP values, while high values resulted in negative SHAP value. A similar pattern was observed for the dinucleotide TA. By contrast, for IMEter, length, G and TC, high feature values generally resulted in a higher SHAP value, with lower values having a negative impact. Notable differences between MDA importance and SHAP importance were observed for the features distance-to-the-TSS and CDS-start, which were considered less important by SHAP, and number of differentially methylated regions (methylation C), which had a stronger effect on model prediction according to SHAP. In the case of C-DMRs, an interesting pattern was observed. While a low number of differentially methylated sites had no effect on the model prediction, high numbers resulted in a negative SHAP value, indicating that the model associated them with lower gene expression. This is consistent with the significantly lower mean gene expression level of C-DMR genes reported above. The low impact of both distance features (distance_TSS, distance_CDS) was yet again surprising, since IME has been associated with both, a short distance to the TSS, as well as being positioned in the UTR. Even more surprising is that very small feature values (distances <200b) were associated with negative SHAP values, i.e. the model was more likely to classify the respective gene as expressed at low levels (Fig. 7d).

**Fig. 8:**
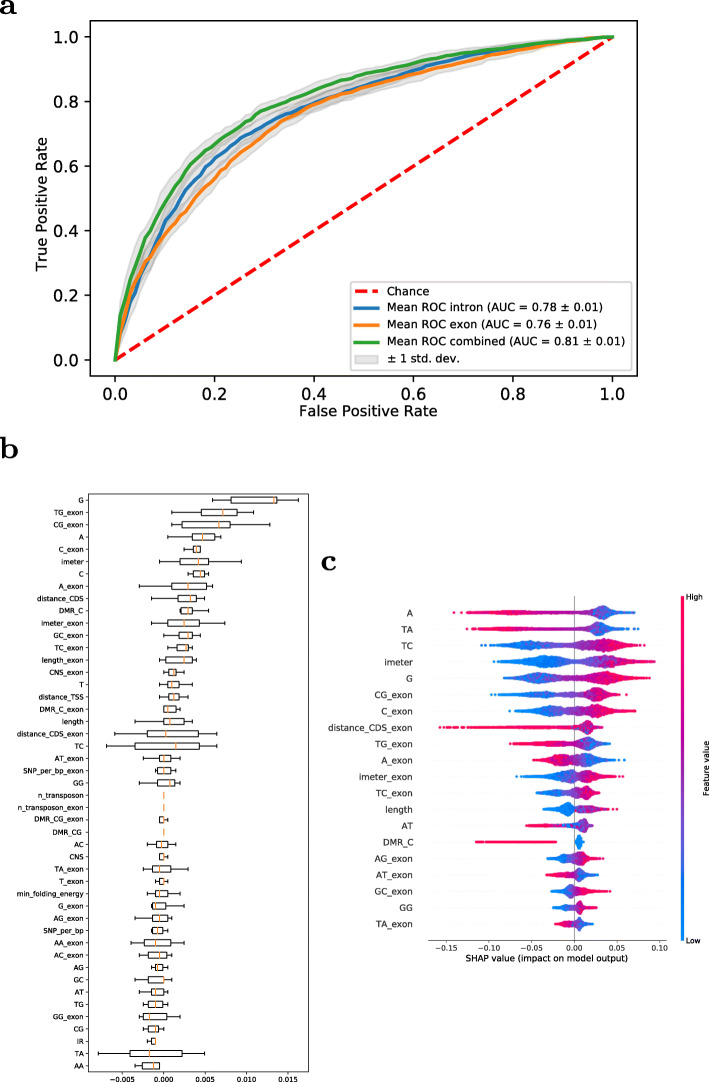
Prediction of gene expression levels based on intron and exon features. Random Forest Performance and Feature Importance. (**a**) Tenfold cross-validated ROC curves for Random Forests trained with intron-only, exon-only features, and both sets combined for the upper/lower quartile data set. (**b**) MDA feature importance for a Random Forest model trained with combined exon and intron features for the upper/lower quartile expression data set. (**c**) SHAP summary plot containing the 20 features with highest importance of the Random Forest model trained with combined exon and intron features for lower and upper quartile dataset. Exon features were extracted from the respective first exons of genes, as were intron-features extracted from first introns

As considered above for the relevance of motifs, it needs to be considered whether there is indeed a specific signal in introns that causes increased gene expression of the associated genes, or whether our classifier simply picks up on features associated with genes that are expressed at high levels, such as housekeeping genes. To test for that, we extracted the same set of features as considered for introns for first exons of the same genes and built RF-models using exon-only, intron-only, and exon-intron-combined features. As shown in Fig. 8a, while the performance of exon-only and intron-only features is comparable (AUC = 0.78), considering both combined leads to a significant increase of predictability (AUC = 0.81). We interpret this as evidence that introns hold information over and above that, which is associated with recognition of highly expressed gene families alone, for which exon-only and intron-only serve as a suitable point of reference. Furthermore, both exon and intron features were considered equally important (Figs. 8b, c).

Taken together, these classification results imply that there is indeed relevant intronic information for determining the expression level class (high vs. low) of genes and suggest a number of informative features.

## Discussion

Intron-mediated enhancement (IME) has been discussed as an important regulator of gene expression, found in nearly all eukaryotic systems tested so far [14] and found associated with highly expressed genes [15, 53, 54]. However, the exact mode of action for inducing expression enhancement is not yet understood. Several different mechanisms have been proposed, with the seemingly biggest open question being the importance of splicing [14]. Several studies suggest that the recruitment of splicing factors, even in the absence of splicing, are the major determinants for the induction of enhancement [14, 16]. Conversely, studies have shown that some introns and intronic motifs were able to induce enhancement irrespective of splicing and splicing factors, and suggested a mechanism acting at the level of genomic DNA [15, 52, 55].

Here, we reported the identification of 16 hexamer and five resulting consensus DNA sequence motifs that may be related to IME in the plant *Arabidopsis thaliana*. Building on previous studies on sequence signals associated with IME [20, 21], our study exploited the available deep sequencing information of more than one thousand *Arabidopsis thaliana* accessions allowing us to probe for conservation as a hallmark of functional relevance. Furthermore, we imposed more explicitly that motifs may show positional preferences within introns, an assumption that appears supported by prior findings [25, 50], and tested as evidence of a functional effect that motif harboring genes exhibit correlated gene expression in addition to elevated expression level, making use of the plethora of available expression information. Thus, we postulated that IME not only leads to increased expression, but also includes a regulatory component, leading to concerted gene expression of subsets of genes. In line with this assumption, out of 81 motifs, identified based on conservation and evidence of preferential intron locations alone, 71, i.e. almost all, were associated with increased correlation (positive, rather than negative effect size, p_bionial, prior = 0.5_ = 1.8E-12, Supplementary Table S1) and 16 (19.7%) were found associated with significantly elevated co-expression (effect size, Cohen’s d > 5%) (Table 2).

In addition to this indication of an existing common regulatory control, we also showed that the identified intron motifs cause elevated gene expression levels. This was tested by exploiting the natural sequence variation across hundreds of Arabidopsis accessions along with available expression information. Allelic variants containing mutated, and thus presumably less active or even inactive motif versions were found to be expressed at lower levels compared to variants of that same gene in different accessions with the unmutated motif version present (Fig. 6, Table 4). While this effect was small and, except for one motif (“AAATCG”, p_FDR_ = 0.04), not significant at individual motif level after correcting for multiple testing, across all candidate motifs, significance was established (*p* = 0.029). Furthermore, at the significance level of FDR < 10%, nine motifs were found significant (Table 4). We consider this result remarkable, as across different accessions, mutations will not only affect our candidate motif - if that were the case, testing for functional relevance would be methodologically cleanest - but many other sites as well, including other gene expression regulatory regions (promoter, enhancers). Furthermore, alleles with variant motifs in their intron may also contain non-mutated motif versions, buffering the mutation effect in another motif instance. As this would further decrease the already low number of genes available for testing, we did not include checking for this possibility. Thus, the observation that despite these additional influences being present, a statistical signal was determined can be seen as strong evidence in support of the functional relevance of the candidate motifs identified here. In this analysis, a stronger signal was observed for the 16 individual hexamer motifs as compared to the consensus motifs (Fig. 6, Table 4). As a consensus motif, by definition, already captures some level of sequence variation, departures from it are more likely to be tolerated and likely less associated with expression effects.

Contributed by the seminal studies on IME [20, 21], IME has been associated with whole introns (IMEter tool and score) and two motifs (CGATT and TTNGATYTG, with the first being a sub-motif of the other) have been implied as functional. Our study enlarges this set by 16 hexamer and five consensus motifs that now can be explored further and experimentally characterized. The IMEter tool has been shown to be a good indicator of IME, with experimentally identified IME introns having consistently high IMEter scores, and the level of enhancement of known IME introns correlating with IMEter score [17, 20, 52, 56]. The motifs identified here and based on conservation, relative occurrence, and positional distribution were comparable with regard to their effect on correlation of gene expression and expression level to genes with the highest IMEter score (Figs. 5b, c). Therefore, it seems likely that the discovered hexamer and consensus motifs are truly related to IME. Building on conservation using intra-species sequence variation, as done here, also is supported by previous observations indicating that regions with high IMEter score were conserved among different species [20, 57].

As the mode of action of IME still is unknown, the fact that we did succeed in identifying motifs that are, based on our filter and test criteria, associated with IME, suggests that either a molecular recognition event - such as binding by proteins - may be at work, or that the motifs play a RNA-structural role relevant for splicing. At this point, using the approaches presented here, we cannot interpret the data in favor of either of the two alternatives. However, our study provides novel candidates for targeted follow-up studies.

In addition to a search for sequence motifs, we performed a Random-Forest-based classification of genes with regard to gene expression level. Here, the goal was to a) prove predictability of expression level using intron-based information, and b) to identify additional features relevant for IME. Indeed, we were able to show that introns hold information on expression level over and above the information provided by the gene context (exon-related information), (Fig. 8a). Overall, base compositional features (most significantly, G-contents) were found most informative, and more important than other parameters such as distance to the TSS or other parameters that would allow to arrive at more interpretable conclusions with regard to mode of action of IME (such as DMRs, folding energy and others) (Figs. 7b, 8b). Low A- and high G-content of the first intron were pivotal for classification as a highly expressed gene. In contrast to the k-mer-based IMEter score, A- and G-content are more general features, describing the composition of the intron and the pre-mRNA. This could indicate a motif-independent influence of first introns on gene expression. Studies have shown that intron composition can regulate splicing by influencing pre-mRNA folding around the splice sites [58], which could explain the observed effects. Compositional effects have also been reported to influence mechanical properties of genomic DNA, such as bending flexibility [59]. However, initial attempts by us to use machine learning to associate reported sequence-dependent DNA flexibility measures to IME proved unsuccessful (not shown). However, considering mechanical properties of pre-mRNA may offer a fruitful avenue for further research. Of note, we checked for GC-content of first vs. other introns and found no relevant difference (G + C content (fraction), first introns: 0.315 +/− 0.051 (s.d), others: 0.318 +/− 0.047).

Confirming the validity of prior approaches, the previously published IMEter score was among the most important features. Correlation between IMEter score and enhanced gene expression by IME has been established, also experimentally for selected gene sets [20]. The results of this study show that this also applies to the whole genome.

DNA-methylation has been shown to play a central role in gene expression regulation, to be linked to nucleosome positioning, and exon-intron boundaries [60] as well as to influence alternative splicing [61]. Regarding the role of DNA-methylation in IME, more specifically, differential methylation (DMRs), given the data and approaches used here, no consistent picture emerged. While C-DMR regions in introns were found associated with increased correlation of the corresponding genes, they were expressed at low levels. Conversely, CG-DMR intron genes showed higher expression, but low correlation. Hence, a cis-regulatory role of DMRs in introns related to IME appears unlikely.

With regard to intron-related cis-regulatory functions, first introns (5′-most) have been considered most relevant [62]. Our observation that first introns, on average, show a slightly increased SNP-density compared to the remaining introns (Fig. 2) appears counter-intuitive. However, introns located in 5′-UTRs exhibit a reduced SNP-density, and hence increased conservation. Therefore, UTR-located introns may play a different functional role than introns embedded in coding regions, which is consistent with previous reports that several UTR introns have been reported to induce IME [62].

Also, when considered as a predictive feature, the distance of the intron to the transcription start site (TSS), with close distances having been discussed as more associated with IME, was not found to be particularly informative. This relatively low impact of the distance to the TSS on the expression level prediction (Figs. 7b, c) is surprising. Previous studies had suggested that proximity to the TSS is an essential property of IME introns [63], and that IME effect declines with distance to the TSS [21]. Our observations were to some degree contradictory. While large distances of introns to the TSS had very little impact on the prediction accuracy of the model, distances shorter than 200 bp increased the likeliness of a gene to be classified as expressed at low, not high levels (Fig. 7d). Parra et al. (2011) observed a similar pattern when comparing the IMEter score of introns to their distance to the TSS. Relatively low IMEter scores were found for introns close to the TSS, with the highest IMEter scores observed at a distance of about 200 bp [20]. However, in their analysis, the observed IMEter scores were still positive, suggesting enhancement, while in the case of the Random Forest model, very small distances were an indication of low expression (Fig. 7d). Our findings suggest that the distance of the first intron to the TSS, as such, is perhaps less important than previously thought, and TSS-proximal introns must, in addition, exhibit a particular composition to lead to IME. The sharp drop in SHAP values, even into the negative value range, for very small distances to the TSS (Fig. 7d), which suggests low expression, may perhaps also indicate gene annotation problems, which need to be inspected on a case-by-case basis.

On the technical side, with regard to the employed gene expression data, this study made use of the large microarray-based dataset covering ~ 20 K genes and thousands of different conditions in order to discern correlated gene expression. As RNAseq has increasingly become de-facto standard, we checked whether consistent results would have been obtained had this study been performed with available RNAseq datasets. Using TravaDB [64], a large compendium of *Arabidopsis thaliana* RNAseq data (158 conditions), we determined a high correspondence of gene expression level (r = 0.88, Supplementary Fig. S2), and, as reported previously [65], also a high correspondence of pairwise correlation (r = 0.49). It should be noted that the probed conditions were very different. Hence, expression level and pairwise correlation proved robust, reflecting condition-independent, coherent expression regulation. Thus, as expression level and pairwise correlation were the two criteria tested for in this study, the microarray data used here can be considered representative.

We performed our analysis within one species (*Arabidopsis thaliana*) with sequence variations amounting to single nucleotide polymorphisms (SNPs). While this intra-species approach eliminates the alignment challenges associated with inter-species studies, evolutionary conservation is confined to a relatively short divergence time (about 5mya [66]). The associated limitations have been discussed before in a study on promoter elements [25] and correlated mutations [67] and apply here as well.

When probing for effects of intron-located motifs on gene expression, the question of expressed alternative splice-forms needs to be considered. We based our analysis on unique first introns (*N* = 24,763). With regard to gene expression and classification, all values were taken as reported and as integrated per gene, not per splice variant. This is due to technical limitations as the used microarray expression data are by design largely splice-variant insensitive. Also, in RNAseq, mappings of short reads (100 bp) to long mRNAs do not necessarily cover relevant splice junctions and the dataset used here does report expression per gene, not per variant, only. However, given the data used in this study, the discrepancy of the gene vs. unique intron seems small (24,763 unique first introns and 21,420 intron-containing genes). Ignoring splice variation can be seen as probing for the relevance of motifs as present in the genome sequence, which may exert their effects on all different splice variants. Thus, the pursued approach may have a precise merit and interpretation For future studies, however, it seems worthwhile to test for specific effects on transcripts emerging from splicing of the respective intron, i.e. those transcripts that originated from a nascent pre-mRNA containing that intron.

Concerning the employed classification methodology, we employed Random Forest classifiers. While recently, deep learning architecture (Recurrent and/ or Convolutional Neural Networks (RNNs, CNNs), have proven to be powerful sequence-based classification approaches [68], RFs, in addition to being a powerful classification engine, allow for a more direct assessment of feature importance, which specifically was the goal of our study.

## Conclusions

Exploiting deep sequencing and broad gene expression information and on a genome-wide scale, this study confirmed the regulatory role on first-introns, characterized their intra-species conservation, and identified a set of novel sequence motifs located in first introns of genes in the genome of the plant *Arabidopsis thaliana* that may play a role in inducing high and correlated gene expression of the genes harboring them.

## Supplementary Information

**Additional file 1: Supplementary information**. Identification of cis-regulatory motifs in first introns and the prediction of intron-mediated enhancement of gene expression in *Arabidopsis thaliana*. **Supplementary Data File**. tar-file of all genes containing the five consensus and two IMEter motifs in their first intron (gene_lists_motifs.tar.gz). **Supplementary Table 1**. Set of 81 hexamers with potential regulatory function as evidenced by increased conservation and positional preferences. ‘Cohen’s d correlation’ is the effect size of difference in the distribution of correlation coefficients between the expression levels of genes harboring the motif relative to a gene set containing frequency-matched random hexamer motifs across all experimental conditions present in the expression dataset. ‘Cohen’s d expression level’ refers to the effect size related to expression level of genes containing the respective motif in the first intron relative to all other intron-harboring genes. Listed also are the numbers of genes, in which the respective intron motif was found. Highlighted bold are the 16 hexamers with Cohen’s d (correlation) > 0.05. **Supplementary Fig. 1**. Comparison of resulting effect sizes (Cohen’s d) when comparing the 81 candidate motifs to count-matched random k-mers (hexamers) as opposed to comparing them to the complete set of other genes. Results were largely consistent (r = 0.83). **Supplementary Fig. 2**. Correspondence of mean expression level of the 20,807 Arabidopsis genes present on both expression platforms (ATH1 microarray and TravaDB-RNAseq). Plotted are the mean expression values across all available conditions in the two databases, respectively, with *N* = 5295 for the microarray set, and *N* = 158 for TravaDB, Pearson correlation coefficient, *r* = 0.88.

## Data Availability

All relevant data are made public as part of this publication or are publicly available.
