# Supplementary material for: Identification of cis-regulatory motifs in first introns and the prediction of intron-mediated enhancement of gene expression in Arabidopsis thaliana
Source: BMC Genomics. 2021 May 26;22:390. doi: 10.1186/s12864-021-07711-1 (PMC8157754; doi:10.1186/s12864-021-07711-1)
Supplement: Supplementary file 1 — Additional file 1: Supplementary information. Identification of cis-regulatory motifs in first introns and the prediction of intron-mediated enhancement of gene expression in Arabidopsis thaliana. Supplementary Data File. tar-file of all genes containing the five consensus and two IMEter motifs in their first intron (gene_lists_motifs.tar.gz). Supplementary Table 1. Set of 81 hexamers with potential regulatory function as evidenced by increased conservation and positional preferences. ‘Cohen’s d correlation’ is the effect size of difference in the distribution of correlation coefficients between the expression levels of genes harboring the motif relative to a gene set containing frequency-matched random hexamer motifs across all experimental conditions present in the expression dataset. ‘Cohen’s d expression level’ refers to the effect size related to expression level of genes containing the respective motif in the first intron relative to all other intron-harboring genes. Listed also are the numbers of genes, in which the respective intron motif was found. Highlighted bold are the 16 hexamers with Cohen’s d (correlation) > 0.05. Supplementary Fig. 1. Comparison of resulting effect sizes (Cohen’s d) when comparing the 81 candidate motifs to count-matched random k-mers (hexamers) as opposed to comparing them to the complete set of other genes. Results were largely consistent (r = 0.83). Supplementary Fig. 2. Correspondence of mean expression level of the 20,807 Arabidopsis genes present on both expression platforms (ATH1 microarray and TravaDB-RNAseq). Plotted are the mean expression values across all available conditions in the two databases, respectively, with N = 5295 for the microarray set, and N = 158 for TravaDB, Pearson correlation coefficient, r = 0.88. [file 12864_2021_7711_MOESM1_ESM.pdf]

Supplementary information

Identification of cis-regulatory motifs in first introns and the prediction of intron-mediated enhancement of gene expression in Arabidopsis thaliana.

Georg Back and Dirk Walther

Supplementary Data File. tar-file of all genes containing the five consensus and two IMEter motifs in their first intron (gene_lists_motifs.tar.gz).

Supplementary Table 1. Set of 81 hexamers with potential regulatory function as evidenced by increased conservation and positional preferences. 'Cohen's d correlation' is the effect size of difference in the distribution of correlation coefficients between the expression levels of genes harboring the motif relative to a gene set containing frequency-matched random hexamer motifs across all experimental conditions present in the expression dataset. 'Cohen's d expression level' refers to the effect size related to expression level of genes containing the respective motif in the first intron relative to all other intron-harboring genes. Listed also are the numbers of genes, in which the respective intron motif was found. Highlighted bold are the 16 hexamers with Cohen's d (correlation)>0.05.

Hexamer	Cohen's d, Correlation, comparable, random hexamer	Cohen's d, Expression level	number of genes
**AGATCG**	**1.45E-01**	**4.60E-01**	**1807**
**ACCCTA**	**9.82E-02**	**1.80E-01**	**2964**
**TCGATC**	**9.16E-02**	**3.40E-01**	**2014**
**TCGGAG**	**8.58E-02**	**2.68E-01**	**857**
**TCTCGC**	**8.13E-02**	**1.95E-01**	**785**
**GATTCG**	**7.68E-02**	**3.23E-01**	**2516**
**ATCGAA**	**7.07E-02**	**3.05E-01**	**4188**
**AAATCG**	**7.00E-02**	**2.83E-01**	**4086**
**AATCGA**	**6.88E-02**	**3.05E-01**	**4406**
**TTAGGG**	**6.76E-02**	**1.95E-01**	**2896**
**ATCGAG**	**6.20E-02**	**2.77E-01**	**1773**
**TCTCGA**	**5.79E-02**	**2.24E-01**	**2044**

**CTCTCG**	**5.77E-02**	**2.34E-01**	**1124**
**AAACCC**	**5.33E-02**	**1.78E-01**	**4970**
**TTCTCG**	**5.27E-02**	**1.86E-01**	**2188**
**TTTCGA**	**5.20E-02**	**2.13E-01**	**3866**
AATCAG	4.56E-02	2.63E-01	4809
TCTCCG	4.34E-02	2.46E-01	1162
ATCTAG	3.92E-02	2.23E-01	2599
TCGAAG	3.90E-02	2.09E-01	1618
TCTTCG	3.77E-02	2.11E-01	2219
ACGAAG	3.57E-02	1.69E-01	1706
TCTCTG	3.35E-02	2.06E-01	4603
TCAATC	3.20E-02	2.66E-01	5130
ACGAAA	3.19E-02	1.46E-01	4121
TTTTCG	3.02E-02	1.10E-01	3623
AACCCA	2.99E-02	1.50E-01	3496
AAATCA	2.88E-02	2.36E-01	9597
ATTGAG	2.83E-02	2.17E-01	4010
GAGACG	2.62E-02	2.40E-01	858
ATCAAC	2.58E-02	1.69E-01	4478
TGAATC	2.58E-02	2.52E-01	5582
TGATTG	2.49E-02	2.36E-01	5348
AAAATC	2.45E-02	2.04E-01	9324
TCAGAG	2.43E-02	1.94E-01	2476
TCTCTC	2.37E-02	2.16E-01	5027
AACAGA	2.07E-02	1.87E-01	6203
AGCAAA	2.00E-02	1.66E-01	5542
TGTTGA	1.85E-02	1.79E-01	5024
AATCAA	1.81E-02	2.68E-01	10140
TTGTTG	1.80E-02	1.60E-01	6810
ATCTTC	1.78E-02	1.49E-01	4563
TTCTTC	1.67E-02	1.73E-01	7402
ATCTCT	1.67E-02	2.03E-01	5623

GTGTTG	1.62E-02	1.67E-01	2973
AGAAGA	1.59E-02	1.68E-01	7292
ATCAAA	1.55E-02	2.31E-01	10140
ATCATC	1.49E-02	1.62E-01	4588
ATCACA	1.48E-02	1.49E-01	4666
AACAAC	1.44E-02	1.28E-01	5421
TTGAAG	1.42E-02	1.34E-01	4498
ATTGAT	1.40E-02	1.98E-01	7170
AACACA	1.39E-02	1.42E-01	6670
AAGAGA	1.31E-02	1.28E-01	7252
AAACAG	1.24E-02	1.12E-01	5683
ATTTTG	1.10E-02	1.16E-01	9516
TGTTTG	1.09E-02	1.41E-01	6804
TAAATC	9.21E-03	1.41E-01	5299
AATTTG	9.01E-03	1.50E-01	7984
TCAAAC	8.93E-03	1.67E-01	5518
ACAGAG	8.80E-03	1.48E-01	4230
TGAAGA	7.73E-03	1.49E-01	4959
AAGAAG	7.47E-03	1.20E-01	6529
ATTAGA	6.13E-03	1.15E-01	5160
AACAAT	5.70E-03	9.62E-02	7233
GGAGAG	5.69E-03	1.54E-01	1542
TTTCGG	3.63E-03	1.36E-01	1464
TTGTGA	2.78E-03	1.39E-01	4996
AAATTA	2.24E-03	1.77E-02	8264
TCACTC	2.01E-03	1.26E-01	2108
AAAAAG	1.61E-05	-3.59E-03	8900
TTTGTC	-3.54E-03	4.03E-02	4384
AAAAAA	-5.95E-03	-5.74E-02	10713
AAATTT	-7.09E-03	1.62E-02	6678
ACACAC	-9.71E-03	7.18E-02	3042
TTTATG	-9.85E-03	3.40E-02	6128

AATTTA	-1.23E-02	3.33E-03	7064
AATTAA	-1.84E-02	-7.39E-02	7059
TTAAAG	-2.03E-02	1.58E-02	4718
TTATAG	-2.06E-02	-1.25E-03	3592
ATTAAA	-2.17E-02	-9.11E-02	7078

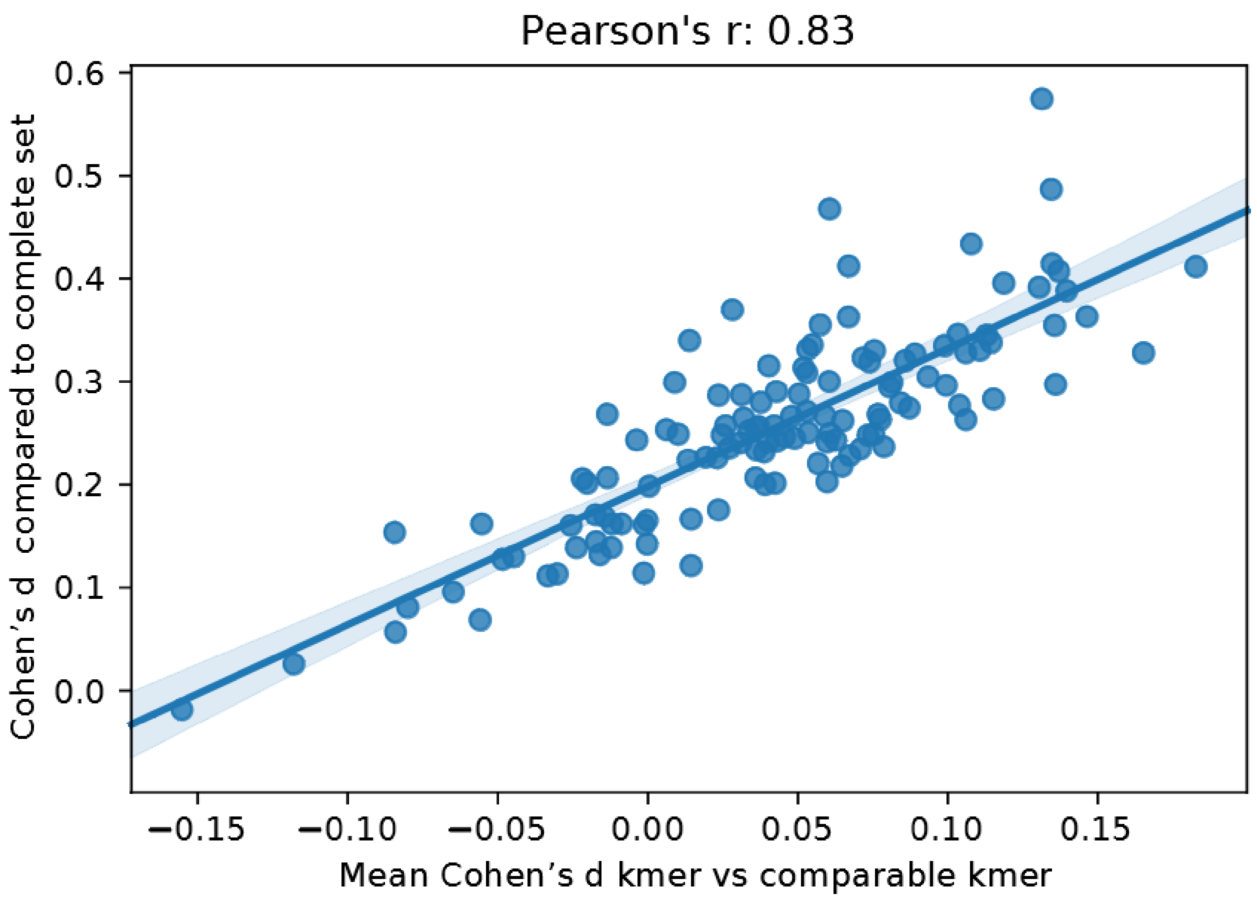

Supplementary Figure 1. Comparison of resulting effect sizes (Cohen's d) when comparing the 81 candidate motifs to count-matched random k-mers (hexamers) as opposed to comparing them to the complete set of other genes. Results were largely consistent (r=0.83)

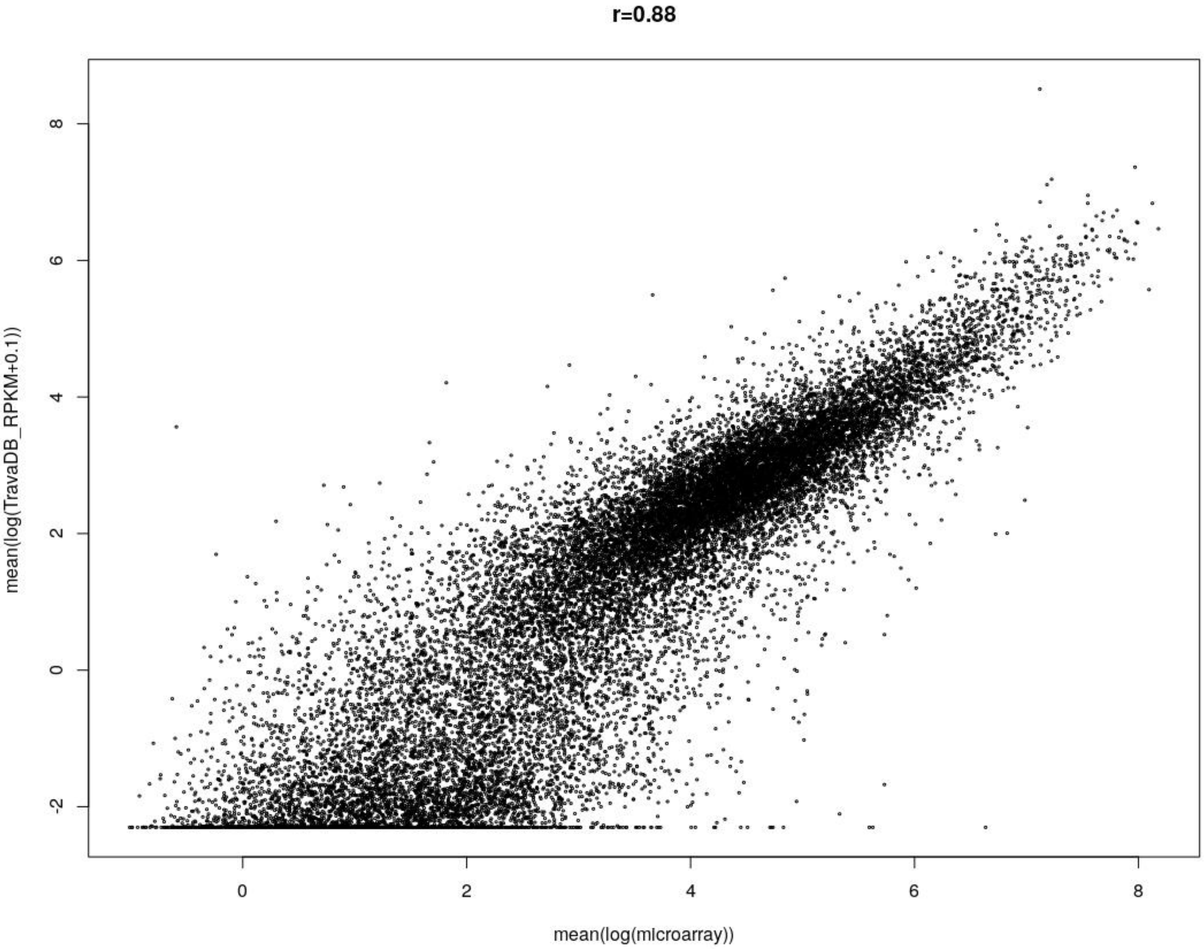

Supplementary Figure 2. Correspondence of mean expression level of the 20,807 Arabidopsis genes present on both expression platforms (ATH1 microarray and TravaDB-RNAseq). Plotted are the mean expression values across all available conditions in the two databases, respectively, with N=5295 for the microarray set, and N=158 for TravaDB, Pearson correlation coefficient, r=0.88.
